# Exploring objective measures for assessing team performance in healthcare: an interview study

**DOI:** 10.3389/fpsyg.2023.1232628

**Published:** 2023-10-24

**Authors:** Rafael Wespi, Tanja Birrenbach, Stefan K. Schauber, Tanja Manser, Thomas C. Sauter, Juliane E. Kämmer

**Affiliations:** ^1^Department of Emergency Medicine, Inselspital, Bern University Hospital, University of Bern, Bern, Switzerland; ^2^Graduate School for Health Sciences, University of Bern, Bern, Switzerland; ^3^Center for Educational Measurement (CEMO) and Unit for Health Sciences Education, University of Oslo, Oslo, Norway; ^4^FHNW School of Applied Psychology, University of Applied Sciences and Arts, Northwestern Switzerland, Olten, Switzerland; ^5^Division of Anesthesiology and Intensive Care, Department of Clinical Sciences, Intervention and Technology, Karolinska Institutet, Huddinge, Sweden; ^6^Department of Social and Communication Psychology, University of Göttingen, Göttingen, Germany

**Keywords:** team performance, objective measures, healthcare, medical simulation training, performance assessment

## Abstract

**Introduction:**

Effective teamwork plays a critical role in achieving high-performance outcomes in healthcare. Consequently, conducting a comprehensive assessment of team performance is essential for providing meaningful feedback during team trainings and enabling comparisons in scientific studies. However, traditional methods like self-reports or behavior observations have limitations such as susceptibility to bias or being resource consuming. To overcome these limitations and gain a more comprehensive understanding of team processes and performance, the assessment of objective measures, such as physiological parameters, can be valuable. These objective measures can complement traditional methods and provide a more holistic view of team performance. The aim of this study was to explore the potential of the use of objective measures for evaluating team performance for research and training purposes. For this, experts in the field of research and medical simulation training were interviewed to gather their opinions, ideas, and concerns regarding this novel approach.

**Methods:**

A total of 34 medical and research experts participated in this exploratory qualitative study, engaging in semi-structured interviews. During the interview, experts were asked for (a) their opinion on measuring team performance with objective measures, (b) their ideas concerning potential objective measures suitable for measuring team performance of healthcare teams, and (c) their concerns regarding the use of objective measures for evaluating team performance. During data analysis responses were categorized per question.

**Results:**

The findings from the 34 interviews revealed a predominantly positive reception of the idea of utilizing objective measures for evaluating team performance. However, the experts reported limited experience in actively incorporating objective measures into their training and research. Nevertheless, they identified various potential objective measures, including acoustical, visual, physiological, and endocrinological measures and a time layer. Concerns were raised regarding feasibility, complexity, cost, and privacy issues associated with the use of objective measures.

**Discussion:**

The study highlights the opportunities and challenges associated with employing objective measures to assess healthcare team performance. It particularly emphasizes the concerns expressed by medical simulation experts and team researchers, providing valuable insights for developers, trainers, researchers, and healthcare professionals involved in the design, planning or utilization of objective measures in team training or research.

## 1. Introduction

### 1.1. Team significance and measures

Collaborative efforts are undeniably essential in providing healthcare. Health-care teams operate in situations that require making high-risk and high-stakes decisions while facing time constraints (Teuma Custo and Trapani, 2020). Empirical research demonstrates that the performance of such teams relies not only on their medical expertise and technical skills but also on their teamwork, that is, their ability to work together effectively (Manser, 2009; Schmutz and Manser, 2013; Schmutz et al., 2019). Furthermore, effective teamwork within health-care teams significantly impacts patient outcomes, as well as staff satisfaction, well-being, and overall organizational success (Heinemann and Zeiss, 2002; Pronovost et al., 2006; Schmutz and Manser, 2013; Rosenman et al., 2018).

Teamwork is a collaborative process in which team members interact and pool their collective resources to meet task requirements such as resuscitating a patient (Fernandez et al., 2008). To ensure high performance, Salas et al. (2008b) have highlighted the importance of various elements of effective teamwork, such as high team cohesion, adaptability, flexibility, and problem-solving skills. However, effective teamwork is often hindered by communication failures, coordination problems, and interprofessional stereotypes (Kozlowski and Klein, 2000; Devine and Philips, 2001; Dietz et al., 2014). Numerous reviews suggest that team trainings and interprofessional education activities can mitigate these obstacles and improve teamwork (Chakraborti et al., 2008; Salas et al., 2008a; Weaver et al., 2014; Fox et al., 2018; Hughes et al., 2019).

In order to identify areas for improvement and to provide feedback to team members in such trainings, it is essential to evaluate team performance in a reliable and valid way (Edmondson, 1999). Establishing appropriate methods for assessing and evaluating team performance is also essential for measuring and monitoring medical teams in their working environment, understanding how to develop and maintain “good” teamwork, and identifying the criteria for “good” teams and outcomes (Jeffcott and Mackenzie, 2008). Measuring team performance is similarly relevant for research purposes, such as when investigating the components of successful teamwork (Murray and Enarson, 2007). However, assessing team performance can be challenging due to the complexity of team dynamics, lack of clear metrics, and limited data and resources (Marlow et al., 2018).

Currently, a multitude of different measures of teamwork is used in the context of team trainings and research. While self-reports and peer assessments are well established, including physiological measures such as the team members’ heart rate variability (HRV) or electrodermal activity (EDA) as indicators of their arousal or stress level are relatively new and still unexplored ways of teamwork assessment. Yet, they deserve a closer exploration as they can potentially mitigate some of the limitations associated with traditional measures such as susceptibility to self-reporting bias (Kozlowski et al., 2013). With our study, we aim to capture the opinions of experts in the fields of medical team training and research on the potentials and challenges associated with integrating physiological and, more generally, objective measures of teamwork into the evaluation of healthcare teamwork. By seeking insights from key stakeholders, this study endeavors to contribute to the theoretical discourse on healthcare teamwork assessment, while also highlighting practical implications for medical training and research.

### 1.2. Traditional and novel evaluation approaches

For a comprehensive evaluation of team performance, it is essential to assess both team processes and outcomes. Team processes include the strategies, steps, and procedures used by the team to accomplish a task (Salas and Cannon-Bowers, 1997). Team performance outcomes focus on results, such as treatment and patient condition. To assess training benefits, medical training studies usually focus on reporting outcomes such as triage accuracy, time to triage, and occasionally administer the perceived benefits from participants (e.g., Luigi Ingrassia et al., 2015; Dittmar et al., 2018; Baetzner et al., 2022). Team researchers typically use the same measures, often assessing time intervals during medical processes, such as decision or execution latency (Burtscher et al., 2011), percentage of hands-on time during resuscitations (Tschan et al., 2009), or durations required to complete a specific task (Tschan et al., 2009). Adherence to institutional standards (Kolbe et al., 2012) or the diagnostic process itself (Tschan et al., 2009) are also considered as measures of teamwork quality.

Traditional data sources for assessing team performance have their advantages and disadvantages (Salas and Cannon-Bowers, 2001; Marlow et al., 2018). Self-reports and peer-assessments can provide access to unobservable reactions, attitudes, and emotions, but may suffer from biases, particularly if individuals are motivated to present themselves in a favorable light. Expert observations based on standardized tools, such as the Team Emergency Assessment Measure (TEAM; Cooper et al., 2010) or Medi-StuNTS (Hamilton et al., 2019), can provide reliable assessment of relevant attributes, but are time- and resource-intensive. On the positive side, measuring these observable behaviors provides actionable guidance for team members to improve their future performance (Rosen et al., 2010).

Research on the unobtrusive measurement of team members’ physiological parameters (biosignals) suggests that an additional source of data can provide valuable information about team processes and unobservable states, such as stress levels, and allow objective assessments of relevant parameters in real time: team physiological dynamics (Kazi et al., 2021; Hałgas et al., 2022). This endeavor is in line with the growing recognition of the multidimensional nature of effective teamwork, highlighting the benefits of considering both visible behaviors and underlying physiological responses (Rojo López et al., 2021). By monitoring physiological signals such as HRV, researchers can assess the arousal, attention, and emotional states of team members during training or real-life scenarios. This information can complement traditional measures to provide a more comprehensive and objective picture of team performance. Moreover, the use of objective measures offers the possibility to shift the focus from an outcome-based assessment toward a process-oriented assessment (Salas et al., 2017; Hałgas et al., 2022). They include specific and measurable data obtained through standardized measurements that are not influenced by personal biases or subjective interpretations. The crucial advantage of objective measures is their ability to capture data at a fine resolution over long periods of time, which cannot be achieved with conventional measures. However, the strategic implementation of objective measures such as physiological data requires a user-friendly methodology that simplifies the analysis and interpretation of data. Furthermore, the collection and analysis of physiological data, for instance, still incurs inherent costs in terms of time and resources.

Despite these potential benefits, the effective use of physiological data in team training and its relationship to higher-order constructs such as successful coordination is still poorly understood. To date, most practical studies in the field of physiological team dynamics have been conducted using simulations of work-related tasks (Kazi et al., 2021; Hałgas et al., 2022). In addition to simulation studies, there are also laboratory studies that investigate physiological team dynamics in video games, simple tasks, or similar (Chanel et al., 2012; Järvelä et al., 2014; Fusaroli et al., 2016). In the medical field, however, there are only a handful of studies, which have investigated only one or two physiological measures like direction of gaze and pupillometry (He et al., 2021) or EDA (Misal et al., 2020). The complexity of the topic and the challenges associated with significant and appropriate implementation could be possible explanations. Therefore, research in this area is essential for the future use of objective data to capture team performance indicators.

### 1.3. Research questions

Our proposed vision is to use objective measures to assess team performance during team training and research to complement traditional team performance assessment. Hereby, objective measures are understood as factual and quantifiable information obtained through standardized measurement and free from personal bias or interpretation, including bio-signals, time stamps, checklists, and the like. By integrating these measures into team assessments, layers of team interactions that often remain concealed may be unveiled, representing new dimensions for the analysis and comprehension of teamwork.

With our study, we aim to contribute to the discussion and ultimately the effective implementation of objective measures into teamwork assessment in training and research contexts. Thereby, we follow the principles of participatory action research, with active engagement of stakeholders who will play a role in its implementation. The primary focus is thus to assess the views of medical team coaches and researchers, key stakeholders in healthcare teamwork, on the viability of integrating objective measures, with particular reference to physiological data, and to identify the potential benefits, challenges and acceptability associated with this approach. We have three main research questions (RQ):

RQ 1: What do experts think about the vision of evaluating team performance with objective measures?

RQ 2: Which objective measures could be used to evaluate medical team performance?

RQ 3: What could be obstacles with the approach of using objective measures to evaluate team performance during team training and research?

In summary, our approach envisions the harmonious integration of objective measures, including physiological indicators, to holistically assess team performance. The aim of this study was to find out what experts in medical education and team research think and know about the opportunities and barriers to evaluating medical team performance using objective measures. In doing so, we aim to provide insights that will shape medical education, research and the wider understanding of teamwork in healthcare.

## 2. Methods

### 2.1. Study design

An exploratory qualitative study design was utilized. We conducted a semi-structured interview study with two expert-groups, followed by a brief on-line survey.

### 2.2. Participants

To answer the three research questions, medical team training and scientific team experts were recruited and interviewed. The inclusion criterion for participation in the interviews was thus either team training expertise as a trainer in medical team training or expertise as a team researcher. Experience in the field of physiological data collection was not required. In addition, we aimed to include an equal number of women and men in the sample.

To identify relevant experts in the field, we used the snowball sampling procedure (Parker et al., 2019). The medical experts were solicited with the help of recommendations from the co-authors, after which they were in turn asked for recommendations at the end of the interviews. For the team researchers, researchers with publications in the field of team research were sought, who were then also asked for recommendations.

### 2.3. Material

The interview questions were developed by the authors in line with the research questions. The complete interview guide can be found in the Supplementary material 1.1. The following analysis will focus on the interview questions from the third block: (a) What comes to your mind when you hear about our vision/goal? (b) Have you considered using objective measures such as biosignals in team training/research to evaluate team performance, and if so, which ones? (c) What are the factors and challenges in assessing team performance using physiological parameters? Where can objective measures be used?

In order to keep the interviews as short as possible, an on-line questionnaire was sent to the interviewees after the interview (via www.soscisurvey.de). It consisted of three questions on age, gender, and expertise (i.e., number of years of experience in training/research context).

### 2.4. Procedure

Identified experts were invited via email to participate in a 30–60 min interview. They were informed that (a) the interview would be recorded, transcribed, and analyzed, (b) their identity would remain confidential, and (c) that participation was voluntary and could be withdrawn at any time.

Interviews were conducted in German or English, according to the preference of the interviewee. At the beginning of an interview, consent was again obtained for the interview to be recorded. The interview procedure was then explained and the interview conducted. Finally, the experts were thanked for their participation, the literature on objective measures of performance was briefly explained, and open questions were answered. They were also offered the opportunity to receive news about the project by email.

After the interviews, participants were sent the on-line survey on demographics. If this was not completed after 7 days, the participants were reminded by email.

### 2.5. Setting

All interviews and questionnaires were collected between June and August in 2022 and were conducted by the first author of this paper. All interviews were conducted using Zoom (Zoom Video Communications Inc., 2016) and recorded using the integrated tool.

### 2.6. Analysis

The transcription of interviews was carried out verbatim by the first author with the help of a speech recognition software (Dragon NaturallySpeaking, Nuance Communications Inc.).

The content analysis was based on the approach of Kuckartz, 2019, which is a rigorous and systematic method used in qualitative research to analyze textual data. It involves the identification and categorization of specific content patterns, themes or codes within the data, providing valuable insights and interpretations for the research study.

All categorizations were carried out by one rater, checked by another rater, and then aggregated into categories by two raters using a consensus procedure. We used MAXQDA 2022.2 (VERBI Software, 2022) for the process of data analysis.

For RQ1 (i.e., opinion on vision), responses were categorized into three categories (positive, neutral, and negative) according to their valence. The positive category included responses that were predominantly positive about the vision presented. The neutral category included all responses that did not have a clear value or where the question was not answered. The negative category included responses where experts expressed a negative or hesitant view such as when they could not relate to the vision or pointed to unsurmountable obstacles.

For RQ2 (i.e., possible measures), responses were categorized based on the measures mentioned. Higher order categories were created to group related measures together such as EDA and electrocardiogram (ECG) together to electrophysiological measures.

For RQ3 (i.e., obstacles), responses were categorized according to the named obstacles identified. Similarly, higher-order categories were established based on the source of these obstacles such as whether they originated from an individual, the model or concerned the implementation.

We decided not to weight the identified categories by their frequency but to treat all responses equally in order to receive a comprehensive overview.

In order to explore how familiar interviewees were with objective measures in the context of team training and research, all responses were examined to determine whether participants had reported personal experience or had undertaken projects or experiments involving objective measures (categorized as “having experience”) or not (categorized as “not having experience).

Demographic data was collected and are presented as means and standard deviations. In addition, *t*-tests were conducted with the software “R,” version 4.3.0, for the variables age and expertise to check whether the two expert-groups differed from each other.

### 2.7. Ethics

The studies involving human participants were reviewed and approved by The Bern Cantonal Ethics Committee (CEC, BASEC Nr: Req-2022-00684). All methods were carried out in accordance with relevant guidelines and regulations. Informed Consent to participate was recorded in writing, at the beginning of the interview and in the survey by each participant. The data were collected, analyzed and stored in pseudonymised form.

## 3. Results

### 3.1. Sample

#### 3.1.1. Demographics

In total, *N* = 34 interviews were conducted (44.1% women, Age: *M* = 48.8 years, *SD* = 11.1, Expertise: *M* = 17.2 years, *SD* = 8.4). One person from the medical group did not respond to the survey. Interviews were conducted with *n* = 21 medical experts who trained medical staff as simulation trainers in Switzerland, Germany, and Austria (38.1% women, Age: *M* = 46.8 years, *SD* = 9.3, Expertise: *M* = 15.5 years, *SD* = 7.2), and interviews with *n* = 13 team research experts conducting research in Europe and the United States (53.9% women, Age: *M* = 51.9 years, *SD* = 13.2, Expertise: *M* = 19.7 years, *SD* = 9.9). The expert groups did not differ significantly from each other in terms of age [*t*(32) = −1.309, *p* = 0.1] and expertise [*t*(32) = −1.435, *p* = 0.080].

#### 3.1.2. Experience with objective measures in simulation training and research

In total, 10 of the 34 experts (29.4%) stated that they had experience with physiological measurements in the context of simulation training and research such as with heart rate or examinations of volume. Of these, eight were from the group of team researchers.

### 3.2. Interview responses

#### 3.2.1. RQ1: experts’ opinion about vision

Of the 34 responses, 19 were positive, 13 were neutral, and three were negative. Both neutral and positive responses were consistently constructive, with curious and skeptical elements. In terms of content, the responses of the expert groups did not differ meaningfully from each other. All three negative responses came from the medical experts.

An example of a positive response:

“[…] In principle, I don't think it's a bad idea. If it were possible to measure stress levels before, during and after a task in a less annoying, less invasive and relatively chic way that would certainly be a good addition to self-reported levels. And I can also see us getting to the point where fitness trackers alone can tell me how well I slept, which means they can certainly tell me how much stress I was under”—(Medical Expert, CM).

An example of a neutral response:

“[…] So what I don't know at the moment is how to do this better in an automated way, although there are certainly people who are extracting data from measurements, whether it's videos, coding or something like that to make it objective but I haven't seen any implementation of that in science yet”—(Research Expert, SK).

An example of a negative response:

“I think it's very challenging, very difficult. It seems very complex and the scenarios are often difficult even for the instructors because you have to be very flexible. Just because we've thought about something in the planning and we know which way it's going to go, doesn't mean it's going to work that way. It is insanely difficult to somehow get a standardized evaluation out of it”—(Medical Expert, DH).

#### 3.2.2. RQ2: possible measures

**Figure 1:**
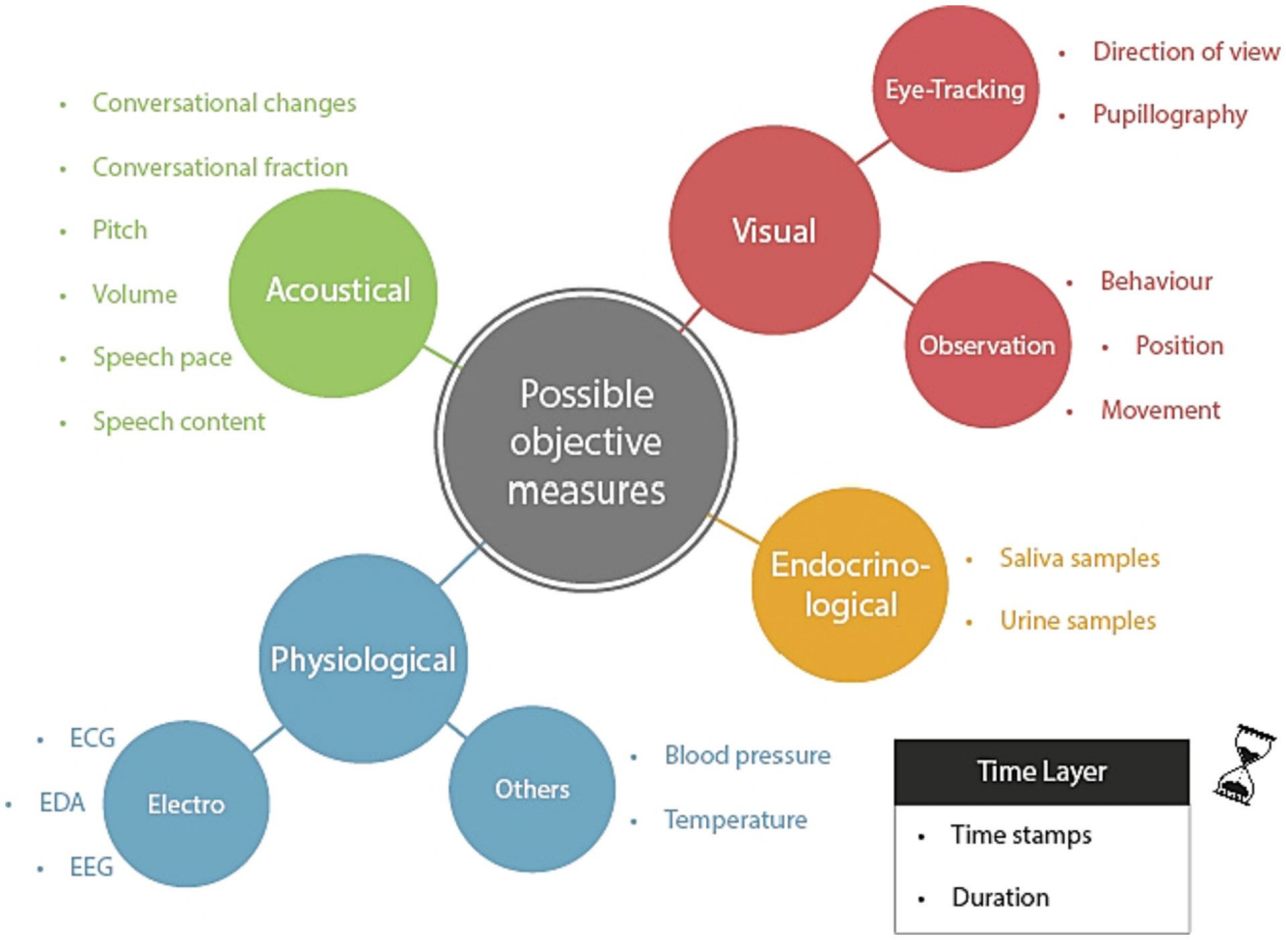
Overview of main and sub-categories concerning the question for potential objective measures. The time layer is to be understood as a meta-layer, which may be integrated with the other layers so that the measured values can be located in their time and duration. EEG, Electroencephalography; EDA, Electrodermal activity; and ECG, Electrocardiogram.

Answers to the second research question were categorized into five main categories: visual, acoustical, physiological, and endocrinological measures, and a time layer (see Figure 1 and Supplementary Table 1 for a list of exemplary answers).

Concerning the time layer, our interview partners highlighted the importance of utilizing time stamps and duration tracking to gain a better understanding of the temporal aspects of social interactions. They emphasized the need for capturing social dynamics in various contexts, particularly in medical settings, and the potential benefits of data-driven approaches for analyzing them.

In terms of physiological data, the experts suggested measuring blood pressure, temperature, electroencephalogram (EEG) signals, and EDA and ECG variables (heart rate and heart rate variability) as potential indicators of stress levels or other relevant factors. Endocrinological measurements, such as analyzing saliva or urine samples, were also named. As they are usually evaluated using laboratory analyses after a training session, rather than in real time, they are depicted as a separate category in Figure 1.

Visual methods named involved observing behavior and movements to indicate the actions taken and the stage of a process. In addition, eye-tracking measures such as pupillography to measure cognitive load, as changes in pupil width can reflect this, and tracking the direction of view to enhance situational awareness and interface design were proposed. Experts acknowledged that careful preparation and interpretation of the data are essential to avoid misinterpretation.

Acoustical measures from the field of communication analysis were suggested to provide useful insights into interpersonal dynamics. These measurements consist of conversational changes that track interaction frequency and nature to assess leadership roles and psychological safety. Conversation fraction analysis captures patterns of interaction and communication frequency among team members. Speech content analysis focuses on the quality of communication. In addition, acoustical indicators like pitch, volume, and speech pace could be studied to comprehend how they impact responses and interactions between individuals.

The list of answers given by the two groups of experts differed only slightly. For example, “pupillography” and “temperature” were mentioned by the medical experts but not by the research experts. On the other hand, the research experts mentioned EEG, which was not mentioned by the medical experts.

#### 3.2.3. RQ3: obstacles

**Figure 2:**
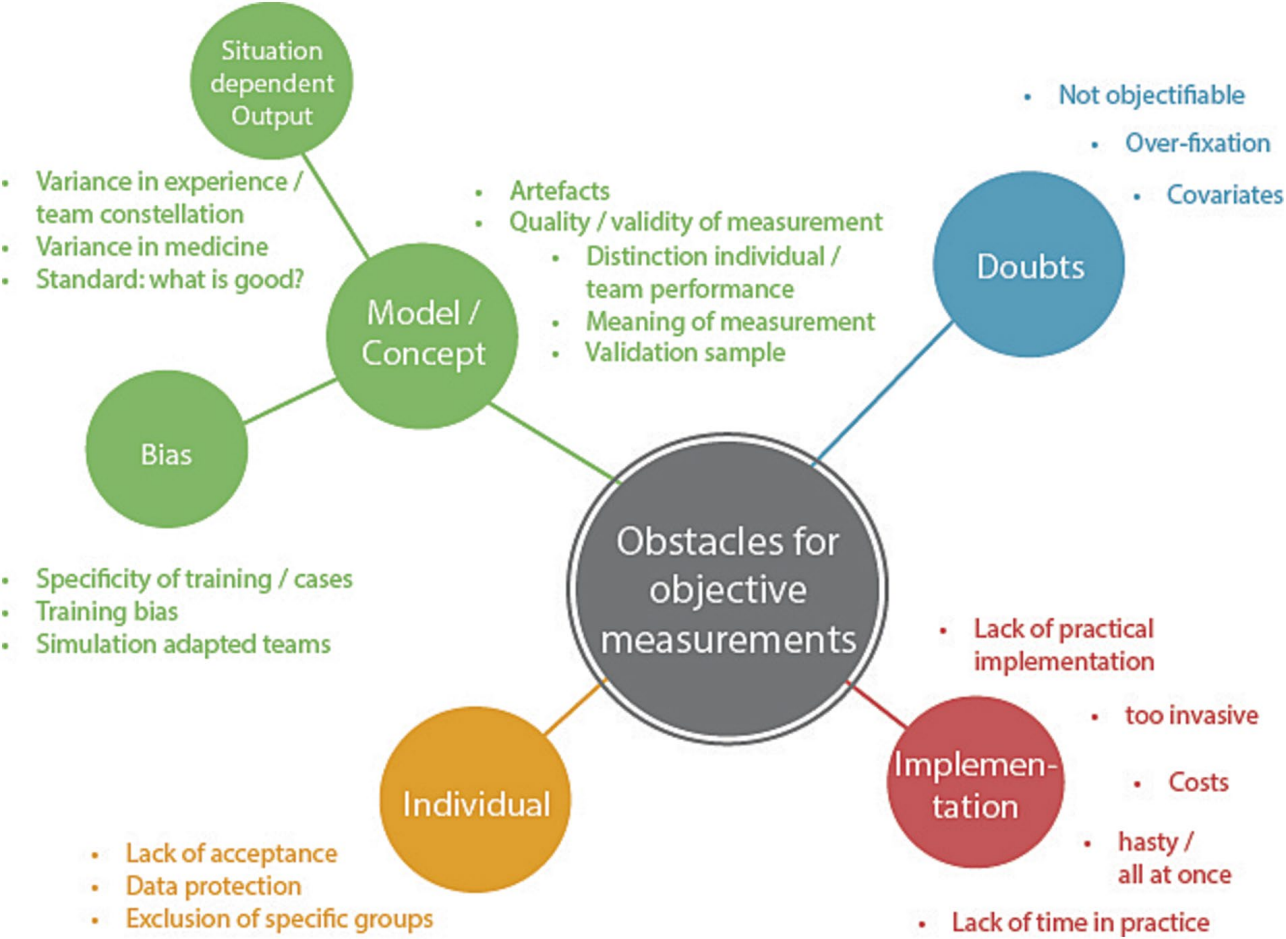
Overview of main and sub-categories concerning the question for potential obstacles of objective measures.

Answers to the third research question were categorized into four main categories: individual, implementation, doubts, and model/concept. The last category consisted of two sub-categories: bias and situation-dependent output. Each category comprised several obstacles, which are summarized in Figure 2.

The interviews disclosed integrated themes that covered the model, bias and situation-dependent output in evaluating the performance of medical teams. Experts acknowledged the importance of artifacts, illustrating how they could both improve and impede training. This encouraged an analysis of the quality and legitimacy of measuring techniques, as well as the difficulty in differentiating individual from collective performance within a convoluted team context. As experts explored the meaning of measurements, they emphasized the importance of validation samples to enhance the reliability of objective evaluation. The discussion broadened to encompass prejudices that arose from training specificity, where particular case requirements and the participants’ consciousness of simulation conditions could influence the outcomes. The experts considered the potential influences of training-induced biases and the impact of team adaptation in simulated scenarios. Moreover, the experts dealt with the situation-specific aspects of output, recognizing the complexities of different medical contexts and the impact of proficiency levels on performance deviation. The efforts to establish the criteria for “good” team performance were emphasized, highlighting the need for flexible measurement standards.

In the interviews, concerns were raised regarding the complete objectification of measuring team performance. Experts acknowledged the complex relationship between factors that influence team dynamics. There were queries about the hurdles of achieving objectivity and separating training effects from a variety of covariates. Worries were expressed about the potential peril of overly fixating on certain metrics, which may overshadow subtle aspects of team interactions. The complexity of assessing and interpreting objective measurements of team performance was recognized, highlighting the multidimensional nature of this subject.

Experts emphasized the delicate balance between obtaining objective data and maintaining the authenticity of the simulation environment. Challenges related to the invasiveness of measurement devices, associated costs, and time constraints were named. Several experts emphasized the importance of a gradual, step-by-step approach to implementation, and ensuring effective navigation of challenges. In parallel, experts recognized the need to address issues related to individual acceptance, data protection, and privacy to ensure the successful integration of objective measurement methods.

The importance of individual factors in measuring the performance of medical teams was identified as a critical theme. Experts acknowledged the need to consider inclusivity and potential exclusions of groups while developing measurement approaches. They considered the complexities involved in ensuring that assessment methods support varied team compositions while accounting for roles and levels of expertise. In addition, they discussed the importance of participants accepting measurement devices and protocols. They raised concerns regarding the potential violation of data protection and privacy while considering the collection of sensitive physiological data. The experts stressed the significance of creating an environment where individual rights and sensitivities are upheld while enabling the thorough measurement of team performance.

Additional obstacles mentioned included technical challenges and biases in the training process as well as the difficulty to distinguish between individual and team performance. To get a more nuanced understanding of the stated obstacles, see Table 1.

**Table 1:** Example answers for each category of obstacles.

	Subgroup	Example
Model/Concept	Artifacts	“There are quite a few artifacts in a simulation training that can change the behavior accordingly, which can be both good and bad for the training.”
	Quality/validity of measurement	“And how is this objective data? Or how can it be collected in real time? How much error is there in collecting it? What the benchmark of the measurement should be and whether it is at an individual level? This is making it difficult to compare individuals within a team.”
	Distinction individual/team performance	“And the question is also what is really teamwork in the sense of individual behavior in the group and how much of the behavior and action is shaped by conflicting goals again within its context?”
	Meaning of measurement	“In the perfect dynamic “online measurement world,” where you see what they see, where they move, what they touch combined with physiological data that connects performance, the question is whether you can make sense of it. So the question is that even if you can collect all this data, can you make sense of it? This is for me one of the biggest hurdles in this regard and should be considered and worked on very carefully.”
	Validation sample	“What comes to mind now is the validation of objective measurements and what that means in terms of significance in the real world. I think that is very important.”
Bias	Specificity of training/case	“You have to accept that teamwork is always very contextual and that you probably cannot say that you always have to do it exactly the same way.”
	Training bias	“And the problem in simulations is that the training participants expect something like this and are prepared to act in such a way, which is absolutely out of touch with reality. Accordingly, they are much more likely to improvise what to expect. In reality, on the other hand, it is so hard to know when to deviate from the procedures because it is a crisis.”
	Simulation adapted teams	“There could be a bias in simulation-adapted teams, which is already known.”
Situation dependent output	Variance in medicine	“This is somewhat difficult in medicine, since there are usually several possibilities and there is usually no absolute correctness, since there is often not one way to solve a problem. As an example in anaphylaxis, that one should deviate from the classic procedure of first placing intravenous access and instead first inject something intramuscularly. That is something that from my point of view the literature is strong enough where the algorithm is also clear. That’s something that’s measurable whether it happens and how fast. That’s the kind of thing you can do well. In some of the other processes or problems, we are a little less clearly structured because the work instructions are also somewhat open-ended.”
	Variance in experience/team constellation	“So let me get this straight, this is extremely complicated, you are going to have a lot of different medical personnel there, with focus on a specific role, with a variety of them and a perceived infinite amount of variance. So I find it exceedingly difficult to measure team performance objectively.”
	Standard: What is good?	“In the end, it boils down to the question of the gold standard, although there are of course other challenges as well. What’s more, performance is currently not very well defined, not to mention not very well discussed.”
Doubts	Not objectifiable	“If we go back to question one, what are the most important things in a team, the question arises whether there are ways to derive these values objectively and how this should be done.”
	Covariates	“I think that it is extremely difficult to find a clear assignment that the training has an effect. Since there are so many things that have an influence.”
	Over-fixations	“I think it would be important to me that you do not shoot down too strongly and that is not the main point in the evaluation. I think we observe a lot as experts and cannot really verbalize why we liked it or not, that’s exactly the development stage from novice to expert. And if I see then only, what key figures from the evaluation have, like so many look contacts for that and so fast until the first support is requested, I could lose myself in these things after.”
Implementation	Lack of practical implementation	“Of course, it is important that the simulation itself is not disturbed. If, for example, the participants had to be completely wired and any bio parameters had to be measured, this would interfere with the simulation. It must also be manageable in the implementation that if you say that a classical simulation is already very complex and if you then have to take very complex measures to determine that, I think that you would not use it so much, because you have to get there first to be able to trust that it also brings something and it has a benefit.”
	Too invasive	“We then realized that this strapping on of ECG cables etc. was already perceived in the study as so invasive that we realized that we could not imagine that in the training context.”
	Costs	“If it is too complex and consumes too much time then it loses a lot of its charm, which would make it very costly and unattractive.”
	Hasty/all at once	“I think that this should be implemented step by step. If you implement this from the beginning with large teams in shock room simulation, you will probably reach your limits relatively soon.”
	Lack of time in practice	“In addition, the time factor is also an important thing, because it must not take significantly longer than usual.”
Individual	Exclusion of specific groups	“The first thing I would add is that you cannot get access for implementation if the teams you want to do it with do not accept it.”
	Lack of acceptance	“In addition, the focus must be on acceptance, so that people can accept the devices for measurement and wear them voluntarily.”
	Data protection	“And you get there into an intimate area of people, which is delicate.”

The list of answers given by the two groups of experts differed only slightly from each other. The points “Bias: simulation-adapted teams,” “hasty/all at once,” “costs,” and “lack of time in practice” were only mentioned by the medical experts, while all other points were mentioned by both expert groups.

## 4. Discussion

The aim of this study was to explore together with relevant stakeholders the potentials and challenges of a novel approach for evaluating team performance for research and training purposes, namely the use of objective measures, by asking experts in the field of research and medical simulation training for their opinions, ideas, and concerns.

To the best of our knowledge, this study is the first to explore the opportunities and challenges of objectively measuring team performance by consulting experts in the relevant fields. Given the increasing feasibility of automated solutions (Kazi et al., 2021; Hałgas et al., 2022), our work thus provides insights to help implement the use of objective measures in the fields of medical simulation training and research, and hints to aspects deemed relevant for the development of such measures from the perspective of its future users.

### 4.1. Reflections on the results

We found that the use of objective measures to assess medical team performance was met with a combination of interest, goodwill, and a degree of skepticism by the participating experts. Responses included a variety of proposed measurement modalities and potential challenges associated with collecting objective data on team performance. Importantly, the responses from both research and medical experts showed a remarkable level of agreement, reinforcing the consistency within the categories and responses identified.

With respect to RQ1 (vision), we found that the approach to use objective measures to evaluate team performance was received largely positively from the experts. However, it must be acknowledged that only a minority of experts had previous practical experience of measuring objective measures such as physiological data. Consequently, the majority of the experts lacked extensive expertise in the specific area under investigation, which is to be expected given the novelty of the topic. It is important to consider this limitation when interpreting the data collected and drawing conclusions from the study.

With respect to RQ2 (measures), experts saw potential in a variety of measurement methods for assessing the performance of medical teams including acoustical, visual, physiological, and endocrinological measures as well as a time layer. All of the listed measures (Figure 1) have already received some attention in research on team performance assessment in different domains (Elkins et al., 2009; Guastello and Peressini, 2017). For example, there is evidence suggesting that team performance correlates with movement patterns (Calabrese et al., 2021) and several physiological measures such as EDA (Pijeira Díaz et al., 2016), ECG (Rojo López et al., 2021), eye-tracking (He et al., 2021), as well as attention (Mahanama et al., 2022). In the majority of these papers, team performance has been inferred using 1–2 measures, although the use of multiple modalities in one measurement would likely add value to the evaluation of team performance (Hałgas et al., 2022). Moreover, most of the existing studies that have attempted to assess team performance using physiological data have focused on simple tasks that may not be directly relevant to medical procedures, and have been conducted under conditions of low movement, which may mask potential artifacts (Stuldreher et al., 2023; van Eijndhoven et al., 2023). Therefore, it is crucial to address these limitations and ensure that future studies properly account for movement and stress artifacts to ensure the validity of performance evaluations.

Various measures have been used to assess not only overall team performance, but also specific elements of team performance, such as shared cognitive load (Collins et al., 2019; Dias et al., 2019; Dindar et al., 2020), shared attention (Stuldreher et al., 2020; He et al., 2021; Pérez et al., 2021), and stress (Cao et al., 2019; Bhoja et al., 2020; Misal et al., 2020). Existing research has largely focused on assessing team performance using measures such as heart rate and EDA, while limited attention has been paid to using motion and voice data for this purpose.

With respect to RQ3 (obstacles), a number of potential barriers to assessing team performance using objective measures were identified. From a research perspective, one of the most relevant obstacles likely is that it is difficult to define a standard for “good” team performance that takes into account the different contexts and preconditions of teams. In fact, the lack of a gold standard for measuring team performance is a widely acknowledged problem (Heinemann and Zeiss, 2002). A standard of team performance should be established via consensus with relevant stakeholders to enable the development and research of objective measures as a solution to this problem. When conceptualizing an objective approach, it is crucial to consider that, depending on the training and its associated learning objectives, various aspects of a teamwork may be emphasized. Thus, a thorough task analysis will be detrimental for establishing standards for “good” teamwork (Tschan et al., 2011).

Another crucial challenge is the question of how to distinguish between team and individual performance, which needs further theoretical work. Challenges specific to the objective measurement approach included the fear of impaired results due to (e.g., movement) artifacts or low measurement quality, which need to be taken into account by developers and users. This concern points to the need for further development and research efforts to optimize the use of physiological data and ensure their reliability and validity in the context of medical team training. Further research is needed to address the remaining obstacles, including the development of a user-friendly measurement process and the establishment of reliable performance assessment models.

From a medical trainer-centered perspective, the most relevant challenges included concerns about trainee privacy and data handling. Ensuring self-determination and privacy were considered crucial for a positive working environment. According to the experts, implementing an objective measurement approach in real-life settings will require considerations of cost effectiveness and smooth integration. Especially limited resources in the health-professions education sector require a simple and reliable measurement system (Maloney and Haines, 2016). Moreover, the approach must be designed such that it is user-friendly and the data output is easily interpretable to generate enhanced values. Therefore, collaborative development of objective indicators along with simulation trainers and medical educators is not only recommended, but also crucial.

### 4.2. Outlook

Objective measures can complement traditional methods and, together, offer a more comprehensive perspective on team performance, although the extent of their impact is currently uncertain. The hope connected with this approach is that objective measures may provide more fine-grained process data and thus enable a greater focus on team dynamics, leading to novel training and research insights. They may thus mitigate shortcomings of, for example, behavior observations that typically result in an average rating per dimension for an entire scenario. Moreover, besides training and research settings, it is possible to gather such data in routine clinical practice to assess team dynamics, improve processes, and identify critical issues. Nevertheless, it is crucial to exercise great caution as this approach should not create any sense of control or supervision among medical personnel at any point.

In particular, training concepts using Virtual Reality (Bracq et al., 2019) provide the opportunity to automatically collect numerous objective measures (e.g., eye tracking-, acoustical-, movement tracking-, and behavior data) without much effort since these sensors are part of a classical head mounted display, which may benefit their training outcomes. Moreover, gathering and integrating physiological data with additional devices are also possible, and unlikely to cause significant disruption during routine simulation training. It is worth noting that virtual, augmented, and mixed reality are relatively new in medical education; nevertheless, they are currently used for this purpose, and extensive research exists that attest to their usefulness and effectiveness in various settings (Barteit et al., 2021; Birrenbach et al., 2021). Consequently, the inclusion of various objective parameters in the assessment of these tools in different contexts is the next logical step. This could also provide benefits for new training and evaluation approaches in the domain of health care education (Collins et al., 2019).

It is important to note that the objective approach to team performance evaluation is not intended to supplement traditional performance evaluation, but rather to focus on the processes and thus enrich the overall evaluation. We acknowledge that each measure alone provides only limited insight into team performance. Therefore, Salas et al. (2017, p. 25) proposed to measure team performance in a comprehensive way by triangulating data in terms of (a) collecting data from diverse sources, including self-reports, peer ratings, and observations, in addition to objective outcomes, (b) measuring performance at the individual and team levels, and (c) measuring both processes and outcomes. Such a triangulation approach also promises a rich basis for debriefing, an essential part of medical team training.

### 4.3. Limitations

Our study comes with some limitations. One limitations is that the majority of the experts we interviewed had no expertise in the specific area of physiological measurement in simulation training or objective assessment using physiological measures. Consequently, their responses were primarily based on subject-specific knowledge or personal beliefs. Yet, the selected experts were key stakeholders in the fields of team research and training and thus representative of the “end users” of objective measures, making it relevant to explore their opinions. It is also worth noting that the field of objective performance assessment, particularly in relation to physiological measures, is still in its infancy and as a result, there are only few experts in this area. To progress, collaboration with experts from relevant adjunct fields is required. Participatory-based model and approach development, based on data, must be continued and consistently improved. Furthermore, it is necessary to involve specialists in the field of measurement technique to prevent issues like unreliable data.

Another limitation is inherent in the interview method, including the potential for respondents to engage in socially acceptable behavior, thereby not fully expressing their true thoughts. To mitigate this risk, participants were assured anonymity to encourage open and honest feedback. Further, it is important to acknowledge that the conclusions drawn from these studies may be limited by the selection of experts (Parker et al., 2019) and the specific questions asked (Halbig et al., 2022).

### 4.4. Conclusion

In conclusion, this study represents an advance in the exploration of objective measures for evaluating medical team performance by providing insights into the opportunities and challenges observed by the relevant stakeholders. The study provides relevant insights for the future development of objective measurement methods in medical simulation training, research, and beyond. Although challenges related to privacy concerns, resource limitations, and complexity may arise, they should be viewed as opportunities for further research and development. Proactively, addressing these challenges will refine and optimize the use of objective measures and provide a robust framework for assessing team performance in healthcare settings. Future research should focus on expanding the scope of physiological data, designing measures with teams, and collecting data to achieve a comprehensive assessment of team dynamics and build a measurement model. By harnessing the potential of objective measures in close collaboration with experts from relevant fields, this study informs future investigations, developments and utilization, ultimately contributing to the advancement of medical education and training practices, leading to improved patient outcomes. However, it is important to note that this is only the first step in a long journey that will continue to rely on close collaboration with medical simulation trainers and team researchers to further develop and implement team assessment using objective measures.

## Data availability statement

The datasets presented in this article are not readily available because consent to share the interviews has not been obtained. Requests to access the datasets should be directed to RW, rafael.wespi@extern.insel.ch.

## Ethics statement

The studies involving humans were approved by The Bern Cantonal Ethics Committee. The studies were conducted in accordance with the local legislation and institutional requirements. The participants provided their written informed consent to participate in this study.

## Author contributions

RW served as the main author and led each part of the working progress. JK and TS made equal contributions in the planning, data acquisition, analysis, and writing stages. TB, SS, and TM contributed to the planning, analysis, and writing steps. All authors contributed to the article and approved the submitted version.
