# Supplementary material for: Exploring objective measures for assessing team performance in healthcare: an interview study
Source: Front Psychol. 2023 Oct 24;14:1232628. doi: 10.3389/fpsyg.2023.1232628 (PMC10628530; doi:10.3389/fpsyg.2023.1232628)
Supplement: Supplementary file 1 [file Data_Sheet_1.docx]

Supplementary Material

Exploring Objective Measures for assessing Team Performance in Healthcare: An Interview Study

Rafael Wespi^1,2*^, Tanja Birrenbach^1^, Stefan Schauber^3^, Tanja Manser^4,5^, Thomas C. Sauter^1†^, Juliane Kämmer^1,6†^

*** Correspondence:**Rafael Wespi, PhD candidate
[rafael.wespi@extern.insel.ch](mailto:rafael.wespi@extern.insel.ch)

# Supplementary Material –

## Interview Script

Thank you for taking the time to do this interview with me. As already mentioned, the aim of this interview is to find out what factors are relevant in team training in order to measure them in a virtual reality (VR) setting using objective signals and to support an evaluation of the team performance as soon as possible / directly after the training. Neither objective features nor VR training should replace the classic team training or the experience of training instructors, instead they should just enhance it and focus on features that are otherwise not simply accessible. In this way, the training could at best be enriched by more detailed feedback in the debriefing.

If you agree, we would record this interview for transcription and content analysis in a further step.

After this interview, we will send you a survey regarding your demographic data, which will be collected as metadata.

All your data will be treated confidentially and will only be used anonymously. In addition, you can withdraw your consent and request the deletion of your data at any time without giving reasons.

- How would you briefly **summarise** **your work/research** in relation to **teams**?

- Out of **your experience**, what makes **a "good" team** in the settings in which you conduct **research[WR2]** ?

         → In the case of difficulties, point out that they can draw from their own experience

→ Specify? 3-5 most important?

- What skills do you want to teach in your team training? Why these in particular?

→ What learning objectives do you aim to achieve in your team training?

- How do you evaluate how well the teamwork went in your team training ?

→ Do you use e.g. standardised observation instruments or self-assessments or similar (global rating, skill elements, etc.) for this?

→ Do you summarise team performance in separate phases or as a global assessment? Do you use other specifics for this?

- Does a debriefing usually take place after your team training?

→ What should be reported back in a team training session so that the participants can benefit from it in the best possible way (e.g. learning objective: Bound? Processible? Usefulness?)?

- What criteria are used to assess the success of your team training sessions?

So far we have talked about the current implementation of team training. I would now like to focus on the future and ask for your ideas and opinions on the question we are currently addressing. We are working on the development of team training using virtual reality technology. Our aim is not to replace classic processes, but to supplement them with technical aids. Furthermore, this approach is to be used in a VR context, which has been rarely done so far (underline visionary part).Our vision is to use biosignals and the data available in VR as soon as possible to improve existing training and to develop new efficient training methods.

- What comes to your mind when you hear about our vision / goal?

- Have you already thought about the use of objective measures such as biosignals that can be collected in a team training session to evaluate team performance?

- In your opinion, what should be taken into account when assessing team performance using physiological parameters and the like?

- Where do you see possible obstacles for an objective assessment of team performance? Where do you think objective measures could be used?

Debriefing:

Acknowledgement and the possibility to receive the transcript of the interview on request.

Follow-up questionnaire by e-mail.

# Supplementary Figures and Tables

## Supplementary Table 1 - Example answers for each measurement category

| **Group** | **Subgroup** | **Example** |
| --- | --- | --- |
| **Time Stamp** | Time stamps | “It occurs to me that there are possibilities to capture the temporal elements that often play a role in the medical context. This could add value in terms of comparability of teams. Of course, with all the advantages and disadvantages that this approach would bring.” |
|  | Duration Tracking | “What I could also imagine is who is in action and how often, or just standing around with nothing to do. Whether that ultimately brings something or not I can't tell you. So whether these behaviours have a certain importance. ” |
| **Acoustical** | Conversational changes | “What I would find exciting would be, for example, the analysis of how often the person communicates or interacts with each other, so for example, if it is a situation so one person should rather take the lead or if we train psychological safety, that as a leader then rather look at all and then ensure that all participate.” |
|  | Conversational fraction | “The interaction between people can be measured, for example, who talks to whom and how much, and when.” |
|  | Speech content | “What I could imagine off the top of my head is how the communication to the patient is when the patient can still communicate, which is very important for us.” |
|  | Pitch | “I think if you could do it with such evaluations, you could also look or analyze how for example the pitch of one person affects another person, what comes back to it or something like that” |
|  | Volume | “I say to look at contexts, why one reacts in such a way, if for example a person was extremely loud or the like around one afterwards to evaluate which led to a reaction or the other way around.” |
|  | Speech pace | “In addition, one could use eye-tracking or speech speed, pitch, speech intensity, movement speed, how loud one is, and the like.” |
| **Visual** | Behaviour | “I think it would also be interesting to look at how handling is carried out, for example how someone holds a VenFlon or performs a discharge or similar, so how this process is carried out in detail and on the basis of this, potential for improvement and improvement measures can be applied.” |
|  | Position / Movement | “What I already saw was the analysis of proximity, that you and I interact with each other in a room, which can be recorded with a sensor more or less well, which notes when we stand in a certain distance to each other that we have an interaction with each other. This is localization data, data for conversations, identification of conversation changes, identification of distance, identification of who opens the conversation and who closes it.” |
|  | Eye-Tracking – Pupillography | “What I once studied, which was a long time ago and which was not in the context of teams, is such pupil response. Because we know that the pupil width or the pupil change can be an indicator for "cognitive load". |
|  | Eye-Tracking - Direction of view | “Especially when it comes to "situation awareness" and so on, to see where people have gone and what we can do in terms of interface design, as well as red posters and frames or something, so that they look there.” |
| **Physiological** | Blood pressure | “...I would think that things like blood pressure, heart rates, electrodermal activity, respiratory rate etc. could be useful for this.” |
|  | Temperature | “I think that's good, if you can find parameters that you can measure, let's just say temperature or whatever, and you can correlate that with leadership measures, I think that's great.” |
|  | EEG | “I think it is possible to measure EEG signals to show where in the brain there is how much activity.” |
|  | EDA | “Skin conductance or things like that can also be surveyed relatively well, but their interpretation is just like with gestures or facial expressions, where there is no objective assignment of meaning.” |
|  | ECG -  Heart rate | “But what I could imagine is, for example, heart rate, that if a person's heart rate goes up it could be a sign of stress or something like that, you would probably have to prepare that very very well, because you can't have a misinterpretation there.” |
|  | ECG -  Heart rate variability | “I can imagine quite a bit of potential there. Then I remember my own studies, where they looked at the 0.1 Hz component of heart rate variability as a potential marker for mental stress, where everyone just wrote that there was no good correlation at all for it.” |
| **Endocrinological** | Saliva samples | “What we have already done, for example, is to collect saliva samples, which is very laborious, however, they are very reliable in such clinical anxiety patients.” |
|  | Urine samples | “Cortisol measurements in urine, although I think it's relatively difficult because of the short half-life and the measurement timing and so.” |

Supplementary Table 1: Example answers for each category. EEG = Electroencephalography, EDA = Electrodermal activity, ECG = Electrocardiography , Hz = Hertz
